# Influence of the Menstrual Cycle on Blood Markers of Muscle Damage and Inflammation Following Eccentric Exercise

**DOI:** 10.3390/ijerph17051618

**Published:** 2020-03-02

**Authors:** Nuria Romero-Parra, Laura Barba-Moreno, Beatriz Rael, Víctor M. Alfaro-Magallanes, Rocío Cupeiro, Ángel E. Díaz, Francisco J. Calderón, Ana B. Peinado

**Affiliations:** 1 LFE Research Group. Department of Health and Human Performance. Faculty of Physical Activity and Sport Sciences. Universidad Politécnica de Madrid (UPM), 28040 Madrid, Spain; n.romero@upm.es (N.R.-P.); beanad16@gmail.com (B.R.); vm.alfaro@upm.es (V.M.A.-M.); rocio.cupeiro@upm.es (R.C.); franciscojavier.calderon@upm.es (F.J.C.); anabelen.peinado@upm.es (A.B.P.); 2 Clinical Laboratory. National Center of Sport Medicine. Health and Sports Department, Agencia Española de Protección de la Salud en el Deporte (AEPSAD), 28040 Madrid, Spain; enrique.diaz@aepsad.gob.es

**Keywords:** sex hormones, creatine kinase, inflammation, female

## Abstract

The aim of this study was to evaluate whether the menstrual cycle and its underlying hormonal fluctuations affect muscle damage and inflammation in well-trained females following an eccentric exercise. Nineteen eumenorrheic women performed an eccentric squat-based exercise in the early follicular phase, late follicular phase and mid-luteal phase of their menstrual cycle. Sex hormones and blood markers of muscle damage and inflammation –creatine kinase, myoglobin, lactate dehydrogenase, interleukin-6, tumoral necrosis factor-α, and C reactive protein– were analyzed in each phase. No effect of menstrual cycle phase was observed (*p* > 0.05), while an interaction for interleukin-6 was shown (*p* = 0.047). Accordingly, a moderate effect size [0.68 (0.53)–0.84 (0.74)], indicated that interleukin-6 values 2 h post-trial (2.07 ± 1.26 pg/mL) were likely to be higher than baseline (1.59 ± 0.33 pg/mL), 24 h (1.50 ± 0.01 pg/mL) and 48 h (1.54 ± 0.13 pg/mL) in the mid-luteal phase. Blood markers of muscle damage and inflammation were not affected by the menstrual cycle in well-trained women. The eccentric exercise barely triggered muscle damage and hence, no inflammation was observed, possibly due to participants training status. The mid-luteal phase was the only phase reflecting a possible inflammatory response in terms of interleukin-6, although further factors than sex hormones seem to be responsible for this finding.

## 1. Introduction

The release of muscle-specific enzymes and proteins into the blood stream is one of the consequences of exercise-induced muscle damage, which is evident from isometric exercise at a long muscle length but it is predominately elicited by eccentric muscle contractions [1]. Muscles may not be accustomed to these exceeding loads which overstretch sarcomeres, and thus, sarcolemma and t-tubules are disrupted [1]. As a result, membrane permeability increases, and calcium enters the cytosol which stimulates some enzymes that may degrade contracting proteins [1]. This contributes to the release of creatine kinase (CK) in the blood stream [2], which has been widely monitored in sports medicine [3], as well as the potential release of myoglobin and lactate dehydrogenase (LDH) [2]. The acute phase of the muscle damage response is characterized by the inflammatory process in response to injury, which facilitates the movement of fluids and cells into the damaged tissue [4]. Essentially, inflammatory cells are involved in the clearance of debris while myogenic satellite cells promote repair and remodeling of tissues [4,5]. The downstream process is triggered by circulating tissue resident leukocytes, among which, neutrophils immediately arrive at the site of injury within the first 12 hours. As a consequence, the production of substances such as reactive oxygen and nitrogen species increases, as well as the secretion of pro-inflammatory cytokines such as tumoral necrosis factor-α (TNF-α) [4,5]. This cytokine— TNF-α—enhances the release of interleukin-6 [IL-6] in response to exercise; however, IL-6 is also released by pro-inflammatory macrophages that arrived at the site of injury to propagate the inflammatory response by secreting other cytokines such as interleukin-1 or interleukin-2 [4,5]. These are the main mediators of inflammation as they attract inflammatory cells and stimulate satellite cell proliferation [4]. However, IL-6 in response to exercise also plays an anti-inflammatory role as it triggers T-regulatory lymphocyte differentiation. Within the first 24 hours, most damaged tissues are phagocytosed, and after 24 hours, anti-inflammatory macrophages and T-regulatory lymphocytes replace the pro-inflammatory cells; therefore, reducing inflammation and enhancing myoblast and differentiation, which promotes the repair of tissues [1]. The release of all these markers into the blood stream and their clearance from plasma also depends on some factors such as participants’ training status, the type, intensity and duration of exercise, and the performance of subsequent bouts of exercise [2,3]. Moreover, the fact that some subjects could be high or low responders, with regards to reporting higher or lower elevations of these serum markers in response to the same stimulus, should be also considered [2]. Finally, the complexity of the female’s hormonal environment due to sex hormones fluctuations throughout the menstrual cycle may be seen as an additional barrier to evaluate muscle damage and inflammation responses to exercise.

Animal studies provide some evidence of estrogens’ protective effects against muscle damage [4,5]. These effects consist of playing an antioxidant role—due to their structure, estrogens facilitate the donation of hydrogens, limit peroxidation chain reactions, and therefore, contribute to membrane stability [6]. In human models, specifically in postmenopausal females, the use of estrogen-based replacement therapies has demonstrated to be beneficial to strength [7], as well as reducing muscle damage and inflammation markers after exercise [8]. However, regarding premenopausal women this effect is not clear. A possible reason could be the complexity of understanding the menstrual cycle and its hormonal fluctuations [9,10]. Estrogen levels vary from their nadirs at the early follicular phase (EFP) coinciding with menses, to its peak at the late follicular phase (LFP) just prior ovulation [11]. After that, estrogen levels fall but moderately rise later in the mid-luteal phase (MLP), when progesterone levels reaches its peak [11]. Thus, such fluctuations should be considered when premenopausal women are evaluated [9].

According to previous research evaluating muscle damage on premenopausal female participants, lower myoglobin or CK responses during high-estrogen phases have been observed [10,12,13,14]. This has been attributed to the presence of estrogen in myofibrils, which may mitigate mechanical damage to muscle fiber membranes; therefore, reducing the release of such proteins into the blood stream, and downregulating inflammation-enhancing genes [10,12,13,14]. In contrast, the lack of differences in muscle damage markers between phases observed in other studies [15,16] have been related to the fact that estrogen concentrations may not differ enough between phases to provide protective musculoskeletal benefits, being therefore estrogen concentration during menses sufficient to offer protection against muscle damage [15]. Further possible reasons of lacking differences could be the individual variation in estrogen concentration between participants or their training status [15]. Nonetheless, most of female studies assessing exercise-induced muscle damage only evaluated one phase of the menstrual cycle due to pursuing different aims such as: comparing eumenorrheic women to oral contraceptive users [17,18], comparing female to male responses [18,19,20], observing responses after different protocols [21] or evaluating the repeated bout effect [22,23]. Therefore, the most important aspect affecting the results from previous studies could be the underrepresentation of the entire menstrual cycle [11], in most cases followed by an incomplete methodology to verify the menstrual cycle phases [24].

On the basis of these controversial findings, the purpose of this study was to evaluate whether the menstrual cycle and the underlying hormonal changes influence blood markers of muscle damage and inflammation, following an eccentric squat-based workout. The squat exercise was chosen considering that it is a functionally relevant movement and a commonly used exercise. Hence, despite being performed in laboratory conditions it could reflect a lifelike training in well-trained females. We hypothesized that muscle damage and inflammation markers present a more pronounced response when concentrations of sex steroid hormones are lower, as in the EFP.

## 2. Materials and Methods

### 2.1. Subjects

Nineteen well-trained eumenorrheic women (28.6 ± 5.9 years of age, 163.4 ± 6.1 cm height, 59.6 ± 5.8 kg body mass, 14.8 ± 5.1 kg fat mass, 42.6 ± 3.1 kg fat free mass, 2.6 ± 0.3 kg bone mineral content) volunteered to participate in this study. Subjects were recruited by means of diverse advertisements published in social media and regional sport competitions from July 2017 to the end of the study. The applicants were filtered through an initial online questionnaire previously fulfilled with personal information and training experience in order to evaluate the following eligibility criteria: (a) to be between 20–40 years old and (b) to be resistance-trained. All participants self-reported their experience in resistance training-completing resistance workouts of about 60 ± 20 minutes, 3 ± 1 times per week, during 5.5 ± 4.9 years- and they were also involved in other sport activities. The exclusion criteria included: (a) irregular menstrual cycles; (b) use of contraceptives in the six months preceding the study, (c) any existing disease and/or metabolic or hormonal disorder; (d) any musculoskeletal injury in the last six months; (e) any surgical interventions (i.e., ovariectomy) or other medical conditions that would be exacerbated by an eccentric resistance exercise protocol; (f) the regular use of medication or dietary supplements that could affect the results; (g) pregnancies in the year preceding; (h) smoking. In order to calculate sample size, the previous results from the study by Sipavičienė et al., [14] were used, which evaluated the main variable of the study (CK) in two different menstrual cycle phases (EFP and LFP). Taking these data as reference, GPOWER software (GPOWER Version 2, Department of Psychology, Bonn University, Bonn, Germany) was used to perform the analysis, suggesting a sample size of 19 to produce a statistical power of 0.80 with an effect size of 0.75 at a significance level of *p* < 0.05. The study consisted of a resistance-based eccentric protocol performed in the three aforementioned menstrual cycle phases (EFP, LFP and MLP) which were adequately randomized and counter-balanced. All procedures complied with the Declaration of Helsinki and the study was approved by the research ethics committee of the Universidad Politécnica de Madrid. A written informed consent was obtained from each participant.

### 2.2. Menstrual Cycle Phase Determination

Eumenorrheic menstrual cycles were defined as regularly occurring menstrual cycles ranging from 24 to 35 days in length [25] in the preceding ≥6 months. Participants confirmed their regular menstruating cycles by providing a retrospective recording of their last six menstrual cycles’ length—with the onset of menses considered as the start of the cycle. Following this information, a gynecologist determined the individual’s different cycle phases and their average phase length, and prospectively estimated the following cycle. To adequately arrange the trials in the different cycle phases, a home urine-based test (Ellatest, Alicante, Spain) was used to detect urine luteinizing hormone (LH). This is a reliable method of predicting ovulation [26] by detecting the LH surge and the subsequent ovulation naturally occurring within the 14-26 hours [9]. The morning mid-stream urine sample was collected on a daily basis in the three to five days prior to the expected ovulation date, previously estimated by the gynaecologist, until LH surge confirmation. Finally, to correctly verify menstrual cycle phases [22], blood samples were collected for the determination of 17-ß estradiol, progesterone, FSH and LH in each phase to confirm that the participants were performing the tests in the correct menstrual cycle phase. In this regard, ovulation was confidently assured when the minimum conservative limit of 16 nmol/L (4.61 ng/mL) of post-ovulatory progesterone was accomplished [11,24].

### 2.3. Screening Protocol 

Participants visited the laboratory within days 2 to 5 of their first menstrual bleeding, at the EFP. In this screening session, blood samples were collected to discard the possibility of an existing disorder. Subsequently, participants’ body composition was analyzed via a Dual-Energy X-ray Absorptiometry (DEXA) scanner (GE Lunar Prodigy apparatus, GE Healthcare, Madison, WI, USA) using the GE Encore 2002 software (v 6.10.029). This screening session concluded with a strength assessment of the lower limbs through a one repetition maximum (1RM) test for the parallel back-squat exercise using a plate-loaded barbell. The 1RM was calculated using an iPhone 6S (Apple Inc., USA) and the Powerlift App [27]. Full range of motion for the lift was always recorded by the same researcher using the Powerlift App [27], with a recording frequency of 240 frames per second. Then, the beginning and the end of the lift was chosen, and the app provided the estimated the 1RM load. According to the manufacturer, these procedures require manual selection by the researcher; thus, two independent observers analyzed the same videos. High interobserver agreement has been shown in previous validation studies (ICC > 0.9) [28].

### 2.4. Eccentric-Exercise Sessions 

**Figure 1:**
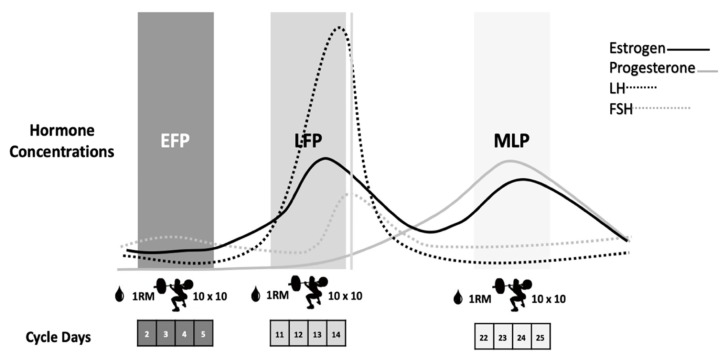
Eccentric-based muscle damaging protocol performed in the EFP, LFP and MLP of the menstrual cycle. EFP: early follicular phase, LFP: late follicular phase, MLP: mid-luteal phase. LH: luteinizing hormone, FSH: follicle stimulating hormone.

Participants repeated the same workout on three occasions according to the EFP, LFP and MLP of the menstrual cycle, as these phases present the most pronounced fluctuations in sex hormones [11] (Figure 1). After a 5-min cycle-ergometer warm-up and some mobility and dynamic stretching exercises, the 1RM for the back-squat exercise was calculated in each session by performing a quick test with the Powerlift App based on the full test previously performed in the screening session. The eccentric-based protocol, which was previously used with resistance-trained males, was carried out in the 3 ± 1 days, 12 ± 3 days and 22 ± 3 days of the menstrual cycle, respectively, for the EFP, LFP and MLP (Figure 1).

It consisted of 10 sets of 10 reps of plate-loaded barbell parallel back squats, at 60% of their 1RM, with 2 min of rest between sets. Squats were performed at a tempo of 4-s eccentric movement, 1-s pause at the bottom, 1-s concentric movement, and a 1-s pause at the top of the lift to focus on the eccentric phase of the lift for greater muscle damage [29]. The tempo was controlled using an interval timer, with the investigator signaling the changes in the lifting phase and giving verbal encouragement. Blood samples were obtained prior to exercise and at 2 h, 24 h and 48 h post-exercise to analyze muscle damage and inflammation markers: CK, myoglobin, LDH, C reactive protein (CRP), TNF-α, IL-6, aspartate aminotransferase (AST) and alanine aminotransferase (ALT).

### 2.5. Blood Sampling and Biochemical Analysis

All blood samples were obtained by venipuncture into a vacutainer containing clot activator. Following inversion and clotting, the blood sample was centrifuged (Biosan LMC-3000 version V.5AD, Riga, Latvia) for ten minutes at 3000 rpm and transferred into Eppendorf tubes and stored frozen at −80 °C until further analysis. Follicle stimulating hormone (FSH), LH, progesterone, 17β estradiol and IL-6 were measured with a COBAS E411 (Roche Diagnostics, GmbH, Mannheim, Germany), using electrochemiluminescence immunoassay (ECLIA) technology. TNF-α was measured with an IMMULITE 1000 system (Siemens Healthineers AG, Munich, Germany) using chemiluminescent enzymatic immunoassay. Finally, CK, myoglobin, LDH, CRP, ALT and AST were analyzed in a Beckman AU400 Clinical Biochemistry analyzer (Beckman Coulter Inc., Brea, CA, USA). Reactive was calibrated following internal laboratory calibration protocols, and controls were assessed after calibration. Coefficients of variation reported by the laboratory were 4.70% for FSH, 5.15% for LH, 4.85% for 17- ß estradiol, 6.35% for progesterone, 4.10% for IL-6, 6.50% for TNF-α, 6.43% for CK, 4.17% for myoglobin, 5.72% for LDH, 6.40% for CRP, 7.19% for AST, and 7.32% for ALT.

### 2.6. Statistical Analysis

Data are presented as mean ± SD. Statistical analyses were conducted using the software package SPSS for Windows, version 25.0 (IBM Corp, Armonk, NY). A Saphiro-Wilk test for normality was used. To analyze hormone concentrations and 1RM in each of the three phases, a one-way ANOVA was performed. To explore our objective, a mixed linear model was performed to analyze the repeated measures, setting phase and time as the fixed effects and subjects as the random effect. Where appropriate, the Bonferroni post hoc test was applied to examine pairwise comparisons for each significant factor. Finally, estimated magnitudes of difference in means and their 95% confidence limits were calculated and presented in standardized units, and were evaluated qualitatively with the following scale: trivial, 0–0.2; small, 0.2–0.6; moderate, 0.6–1.2; large, 1.2–2.0; and very large, >2.0 [30]. Significance level was set at *p <* 0.05.

## 3. Results

### 3.1. Sex Hormones and Strength Assessment

Results for the hormonal analysis confirmed that the trials were performed in the correct menstrual cycle phases. A significant effect of menstrual cycle phase was observed for 17β–estradiol (38.2 ± 32.1, 185.1 ± 173.9 and 156.1 ± 91.5 pg/mL), progesterone (0.3 ± 0.1, 0.4 ± 0.7 and 10.1 ± 3.9 ng/mL), FSH (7.1 ± 2.5, 6.1 ± 2.9 and 3.1 ± 1.2 mUI/mL) (*p* < 0.001 for all variables), and LH (6.0 ± 1.9, 11.9 ± 10.3 and 4.4 ± 2.4 mUI/mL; *p* = 0.008), respectively for the EFP, LFP and MLP. Results for the 1RM were 75.9 ± 16.8 kg, 75.5 ± 17.4 kg and 76.0 ± 16.8 kg for EFP, LFP and MLP, respectively, and reported no differences between phases (*p* > 0.05).

### 3.2. Muscle Damage

**Figure 2:**
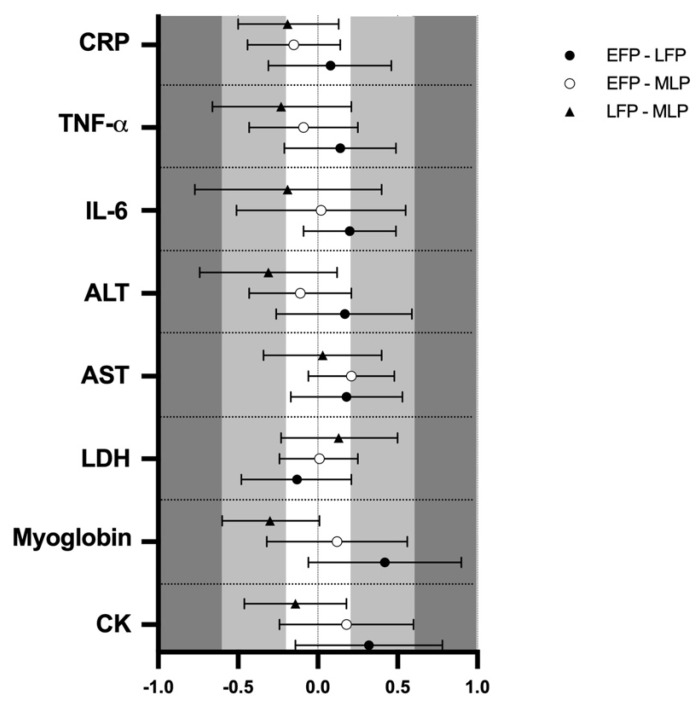
Standardized mean differences (ES) and confidence intervals in muscle damage and inflammation markers among menstrual cycle phases. EFP: early follicular phase, LFP: late follicular phase, MLP: mid-luteal phase. CK: creatine kinase, LDH: lactate dehydrogenase, AST: aspartate aminotransferase, ALT: alanine aminotransferase, IL-6: interleukin-6, TNF-α: tumor necrosis factor α, CRP: c reactive protein.

Muscle damage markers results and pairwise comparisons are shown in Table 1. Results showed no effect of menstrual cycle phase in CK (133.8 ± 12.1, 146.7 ± 12.1 and 138.1 ± 12.1 U·L^−1^) myoglobin (73.2 ± 2.9, 79.6 ± 3.7 and 74.1 ± 2.8 µ·L^−1^), LDH (170.9 ± 3.1, 169.3 ± 4.6 and 170.5 ± 3.1 U·L^−1^) and AST (22.4 ± 1.0, 23.2 ± 1.1 and 23.3 ± 0.9 U·L^−1^) respectively for the EFP, LFP and MLP (*p >* 0.05 for all variables). The analysis of ES revealed low results when comparing menstrual cycle phases (Figure 2). In contrast, a trend for menstrual cycle phase was observed for ALT, (16.3 ± 1.0, 17.3 ± 0.9 and 23.7 ± 0.9 Ui/L; F_2,49_ = 3.067; *p =* 0.056) indicating that in the LFP, ALT values were almost higher than in MLP (*p =* 0.068), but ES was not meaningful (Figure 2). No significant interaction between time and menstrual cycle phase was observed for all these markers and the ES was negligible (data not shown). 

**Figure 3:**
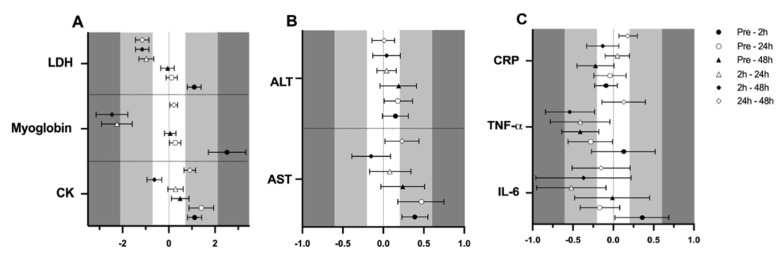
Standardized mean differences (ES) and confidence intervals in muscle damage and inflammation markers among time-point measurements. (**A**)CK, myoglobin and LDH; (**B**)AST and ALT; (**C**)IL-6, TNF-α and CRP; EFP: early follicular phase, LFP: late follicular phase, MLP: mid-luteal phase. CK: creatine kinase, LDH: lactate dehydrogenase, AST: aspartate aminotransferase, ALT: alanine aminotransferase, IL-6: interleukin-6, TNF-α: tumor necrosis factor α, CRP: c reactive protein.

Finally, a significant effect of time (*p <* 0.001 for all variables) was observed in CK (105.0 ± 6.2, 152.4 ± 10.6, 173.8 ± 16.9 and 125.6 ± 9.0 U·L^−1^; F_3,161_ = 37.543), myoglobin (61.1 ± 1.5, 114.2 ± 9.0, 65.2 ± 1.7 and 61.9 ± 1.4 µ·L^−1^; F_3,150_ = 63.747), LDH (164.9 ± 3.4, 184.3 ± 4.1, 167.3 ± 3.6 and 164.4 ± 3.3 U·L^−1^; F_3,154_ = 33.883) and AST (21.8 ± 0.9, 23.4 ± 1.2, 23.7 ± 0.9 and 22.8 ± 1.1 Ui/L; F_3,162_ = 6.514), respectively at pre-trial, 0 h, 24 h and 48 h, supported by the ES (Figure 3A,B). No effect of time was observed for ALT (21.8 ± 1.0, 23.4 ± 0.9, 23.7 ± 0.9 and 22.8 ± 1.1 Ui/L; *p <* 0.05).

### 3.3. Inflammation

Regarding inflammation markers, results are also presented in Table 1. Results showed no effect of menstrual cycle phase in IL-6 (1.7 ± 0.4, 1.8 ± 0.5 and 1.7 ± 0.4 pg/mL), TNF-α (4.8 ± 0.9, 5.0 ± 1.2 and 4.8 ± 1.2 pg/mL) or CRP (0.52 ± 0.07, 0.54 ± 0.06 and 0.47 ± 0.06 mg/L), respectively for the EFP, LFP and MLP (*p >* 0.05 for all variables); while the ES was low for menstrual cycle phase-effects (Figure 2). An interaction between time and menstrual cycle phase (F_6,150_ = 2.192; *p =* 0.047) was observed for IL6. Accordingly, a moderate ES indicated that only in the MLP were 2 h post-exercise values higher than pre-trial [0.68 (0.53)], 24 h [0.84 (0.74)] and 48 h [0.75 (0.75)]. This effect was not observed in the EFP and LFP. No interaction or meaningful ES was observed between time and menstrual cycle phase for CRP and TNF-α (data not shown). A significant effect of time for IL-6 (1.7 ± 0.4, 1.9 ± 0.7, 1.6 ± 0.3 and 1.7 ± 0.3 pg/mL; F_3,154_ = 2.955; *p =* 0.034), TNF-α (5.0 ± 1.3, 5.2 ± 1.1, 4.7 ± 0.9 and 4.6 ± 0.9 pg/mL; F_3,156_ = 3.958; *p =* 0.009) and CRP (0.53 ± 0.06, 0.51 ± 0.06, 0.54 ± 0.07 and 0.47 ± 0.06 mg/L; F_3,159_ = 2.759; *p =* 0.044) was obtained for pre-trial, 0 h, 24 h and 48 h, respectively. A moderate ES indicated that TNF-α concentrations at 2 h seemed to be higher in comparison to 48 h (Figure 3C).

## 4. Discussion

The major finding of this study is that the hormonal environment throughout the different menstrual cycle phases did not affect blood markers of muscle damage and inflammation. The only variable showing a possible exercise-induced inflammatory response was IL-6 in the MLP; although this finding should be taken with caution. This workout based on eccentric squats elicited muscle damage, as could be observed from post-exercise concentrations of CK, myoglobin and LDH. In addition, in a previous study conducted by our laboratory, high post-exercise muscle soreness was also observed in the same sample following an identical exercise protocol (unpublished data). However, this exercise-induced damaging exercise was not strenuous enough to trigger inflammation. This lack of menstrual cycle phase differences in blood markers of muscle damage is potentially due to the fact that our participants were well-trained in resistance training [31]. This is in agreement with a previous study which also obtained no differences between the EFP and LFP in serum CK following a drop jump protocol [16]. They indicated that previous exposures to eccentric exercise can influence the CK response, suggesting that CK concentration is an unreliable marker of muscle damage in training studies where the same muscle groups are exercised in a subsequent session [32]. Another possible explanation could be the high variability in the response to a muscle-damaging exercise. This is associated with the presence of some polymorphisms present in genes encoding for the myofibrillar proteins α-actinin 3 and myosin light chain kinase, which influence CK and myoglobin responses [28]. Altogether, this might indicate that higher estrogen concentrations during LFP and MLP may not provide extra protection against muscle damage in comparison to the EFP as the literature suggests [4,5,11,15].

Regarding the effect of menstrual cycle phase on inflammation response, IL-6 values seem to be higher 2 h post-exercise than at the other time-points, hence indicating a likely inflammatory response to exercise, only observed in the MLP. In fact, from some previous studies indicating that estrogen has a role in the regulation of immunocompetence [33,34,35,36], most of them reported an upregulated pro-inflammatory response to exercise in the luteal phase in comparison to the mid/late follicular phases [35,36]. However, it may possible that these gene expression changes in response to exercise, may represent a swift return to baseline from a highly anti-inflammatory state at rest, as opposed to presenting a truly pro-inflammatory response. Another possible reason may be the presence of progesterone during the MLP which may interact with certain estrogen-protective responses, resulting in an increase in inflammation. This interaction between estrogen and progesterone has been observed in other endocrine responses [37]. Interestingly, the increase in IL-6 at 2 h in the MLP was not preceded by an increase in TNF-α. Therefore, such an increase in IL-6 might be not attributed to the inflammatory response but to the endocrine role of muscle, which enhances IL-6 concentrations after glycogen depletion during exercise [15]. However, on the basis of IL-6 as a myokine, the highest IL-6 concentrations have particularly been observed at the cessation or 30-min post exercise [38,39]. In addition, it should be taken into consideration that TNF-α rarely increases in response to exercise, except for highly strenuous prolonged efforts such as marathons [40]. Thus, the potential role of IL-6 as a myokine could be doubtful, with the data indicating a more likely inflammatory role.

Another factor possibly involved in IL-6 changes may be the modality of exercise. A previous study obtained higher values of IL-6 in the mid-follicular phase in comparison to the MLP after a continuous running protocol [10], while a different study obtained no differences between the EFP and the MLP following a running protocol [15]. As such, more research is needed to clarify whether different exercise modalities could facilitate different increases in IL-6 in relation to menstrual cycle phase. In line with that, our lacking time-differences in IL-6 concentration between pre-trial and 2 h could be also due to the exercise modality. In the present exercise protocol, stimulus duration as well as the muscle mass involved in the exercise may be insufficient to increase IL-6 from pre-trial levels [15]. This has been demonstrated following aerobic protocols [40] which are generally long-lasting and involve larger muscle groups. In fact, our findings revealed that IL-6 and TNF-α concentrations decreased after 24 h in comparison to 2 h independent of the phase studied. This, however, is unlikely to be associated with exercise due to the aforementioned absence of differences between pre-trial and 2 h measurements. Interestingly, despite the lacking inflammatory effect reported, a remarkably moderate effect might suggest reductions in TNF-α at 48 h in comparison to 2 h which could be in agreement with some of the anti-inflammatory effects of exercise, as indicated in the literature [40].

Lastly, menstrual cycle phases do not seem to affect AST and ALT; however, it is challenging to discuss this finding due to the lack of literature evaluating these enzymes during the various menstrual cycle phases. Interestingly, our ALT values appear to be higher in the LFP for all time-points. Although ALT is mainly located in the liver, alanine synthesis also occurs in the muscle [41]. Then, according to these results, participants’ higher estrogen concentrations during the LFP may predispose participants’ muscle tissue to release this enzyme as a consequence of muscle injury due to an exercise overload. However, our values are within normative values and do not appear to be indicative of muscle injury. In fact, ALT increases have been reported following extreme physical exertion such as Thai boxing or ultramarathons [41], so it is very unlikely that our squat-based protocol elicited such a response. It has also been suggested that pro-inflammatory cytokines may exert a catabolic effect on muscle, which would facilitate the increase of ALT [42]. Thus, the higher ALT concentration in the LFP would agree with some results that suggest the lack of estrogen’s protective effect, but further investigation is warranted. On the contrary, the AST response was not affected by menstrual cycle phases but increased in post-exercise measurements, which is in accordance with previous female studies [43,44].

To the best of our knowledge, this is the first study to investigate muscle damage and inflammation responses induced by an eccentric exercise focused on squats, in the same group of participants, and considering the menstrual cycle phases with the most pronounced changes in hormonal concentrations. As far as we are concerned, the current study presents an adequate methodology to verify menstrual cycle phases: hormonal analysis, calendar counting and LH confirmation via urine-based tests. Intriguingly, despite being a robust methodology, and a gynecologist confirmed the regularity of participants’ menstrual cycles, individual variation in muscle damage and inflammation markers was typically highly variable. This may be a confounding factor to observe differences between menstrual cycle phases and the results should be taken cautiously. Additionally, the choice of the exercise protocol may have also been troublesome to elicit noteworthy differences between menstrual cycle phases. Despite this squat-based workout –that could be incorporated into regular female athletes— was previously performed to elicit muscle damage [29], it barely triggered muscle damage, and hence, inflammation response in our sample. The training status of our well-trained participants may have affected exercise-induced muscle damage development. Finally, as a result of this design, previous studies in the literature are not entirely comparable to ours and it could also be considered as a limitation. However, despite the aforementioned drawbacks the findings provided by this study could contribute to elucidate the lacking influence of sex hormones on muscle damage and inflammation, following a squat-based workout potentially usable by coaches on a regular basis. 

## 5. Conclusions

In conclusion, no differences between menstrual cycle phases are observed in blood markers of muscle damage and inflammation following a resistance training focused on eccentric squats. The only phase to report a possible inflammation-related pattern in terms of IL-6 was the MLP. However, the lack of differences in estrogen between the LFP and the MLP make it difficult to relate this finding to the lack of estrogen’s protective effect. Further research should focus on homogenizing phases measured and verification in order to properly clarify the influence of hormonal status on blood markers of muscle damage and inflammation.

## Figures and Tables

**Table 1:** Blood markers of muscle damage and inflammation throughout the menstrual cycle following an eccentric-based resistance exercise. Data expressed as mean (SD).

	EFP		LFP		MLP		TOTAL	
**CK (U** **·** **L^−1^)**								
Baseline	108.6	(48.0)	105.7	(33.1)	100.7	(29.9)	105.0	(37.3)
2 h	151.6	(70.0)	155.1	(44.9)	150.6	(43.8)	152.4 ^*^	(53.3)
24 h	154.1	(69.3)	195.5	(95.3)	172.1	(85.8)	173.9 ^a^	(84.4)
48 h	117.3	(40.1)	130.6	(47.7)	128.8	(49.5)	125.6 ^a,c^	(45.5)
Total	132.9	(60.7)	146.7	(67.7)	138.1	(61.1)		
**Myoglobin (µg** **·** **L)**								
Baseline	62.8	(8.2)	60.4	(7.2)	60.1	(10.6)	61.1	(8.9)
2 h	105.5	(43.9)	129.1	(56.3)	107.9	(41.2)	115.0 ^**^	(48.7)
24 h	64.5	(9.6)	64.9	(7.9)	66.1	(10.7)	65.2	(9.6)
48 h	59.8	(7.4)	63.8	(9.0)	62.2	(8.1)	61.9	(8.5)
Total	73.2	(30.0)	79.6	(41.0)	74.1	(30.1)		
**LDH (U** **·** **L^−1^)**								
Baseline	166.5	(14.7)	164.3	(22.6)	163.9	(13.4)	164.9	(17.4)
2 h	187.1	(20.3)	181.5	(26.8)	184.4	(16.3)	184.3 ^**^	(21.3)
24 h	166.2	(15.9)	170.2	(23.9)	165.7	(17.4)	167.4	(19.1)
48 h	163.8	(16.5)	161.4	(16.2)	167.9	(16.9)	164.4	(16.5)
Total	170.9	(19.1)	169.3	(23.6)	170.7	(18.9)		
**IL-6 (pg/mL)**								
Baseline	1.7	(0.7)	1.7	(0.5)	1.6	(0.3)	1.7	(0.6)
2 h	1.8	(0.7)	1.7	(0.6)	2.0	(1.3)	1.9	(0.9)
24 h	1.6	(0.2)	1.7	(0.7)	1.5	(0.0)	1.6 ^b^	(0.4)
48 h	1.6	(0.5)	1.9	(0.7)	1.5	(0.1)	1.7	(0.4)
Total	1.7	(0.5)	1.7	(0.6)	1.7	(0.7)		
**TNF-** **α** **(pg/mL)**								
Baseline	4.9	(1.1)	5.3	(1.9)	4.9	(2.0)	5.0	(1.7)
2 h	5.2	(1.4)	5.3	(1.6)	4.9	(1.4)	5.2	(1.5)
24 h	4.8	(1.4)	4.8	(1.0)	4.6	(0.9)	4.7 ^##^	(1.1)
48 h	4.5	(0.9)	4.6	(0.9)	4.7	(1.2)	4.6	(1.0)
Total	4.8	(1.2)	5.0	(1.4)	4.8	(1.4)		
**CRP (mg/L)**								
Baseline	0.6	(0.3)	0.6	(0.3)	0.5	(0.3)	0.5	(0.3)
2 h	0.5	(0.3)	0.5	(0.3)	0.5	(0.3)	0.5	(0.3)
24 h	0.6	(0.4)	0.5	(0.3)	0.5	(0.3)	0.5 ^##^	(0.3)
48 h	0.5	(0.3)	0.5	(0.3)	0.5	(0.3)	0.5 ^b,#^	(0.3)
Total	0.5	(0.4)	0.5	(0.3)	0.5	(0.3)		
**AST (Ui/L)**								
Baseline	21.2	(4.4)	21.8	(5.0)	22.6	(5.1)	21.8	(4.8)
2 h	22.8	(4.2)	23.4	(4.7)	23.8	(4.2)	23.4 ^a^	(4.3)
24 h	23.1	(4.7)	24.3	(5.1)	23.8	(4.0)	23.7 ^a^	(4.6)
48 h	22.4	(5.2)	23.3	(5.4)	22.8	(4.6)	22.8	(5.0)
Total	22.4	(4.6)	23.2	(5.0)	23.3	(4.5)		
**ALT (Ui/L)**								
Baseline	16.0	(4.5)	16.8	(5.5)	14.7	(4.9)	15.8	(5.1)
2 h	16.3	(4.4)	17.3	(5.4)	15.6	(4.1)	16.4	(4.7)
24 h	16.4	(4.5)	17.4	(5.3)	15.8	(3.9)	16.6	(4.6)
48 h	16.4	(5.1)	17.5	(5.9)	15.9	(4.4)	16.6	(5.1)
Total	16.3	(4.5)	17.3	(5.4)	15.5 ^§^	(4.4)		

EFP: early follicular phase, LFP: late follicular phase, MLP: mid-luteal phase, CK: creatine kinase. LDH: lactate dehydrogenase; IL-6: interleukin-6; TNF-α: tumor necrosis factor; CRP: C reactive protein; AST: aspartate aminotransferase; ALT: alanine aminotransferase; ^*^ Different from the rest of time-points (*p* < 0.05); ^**^ Different from the rest of time points (*p* < 0.001); ^a^ Different from pre-trial (*p* < 0.05); ^b^ Different from 2 h post-exercise (*p* < 0.05). ^c^ Different from 24 h post-exercise; (*p* < 0.05). ^#^ Trend is different from pre-trial (*p* = 0.059); ^##^ Trend is different from 2 h (*p* = 0.064 and *p* = 0.084, respectively, for TNF- α and CRP); ^§^ Trend is different from LFP (*p =* 0.068).
